# Broad spectrum antibiotic-degrading metallo-β-lactamases are phylogenetically diverse

**DOI:** 10.1007/s13238-020-00736-4

**Published:** 2020-06-15

**Authors:** Marcelo Monteiro Pedroso, David W. Waite, Okke Melse, Liam Wilson, Nataša Mitić, Ross P. McGeary, Iris Antes, Luke W. Guddat, Philip Hugenholtz, Gerhard Schenk

**Affiliations:** 1 School of Chemistry and Molecular Biosciences, The University of Queensland, St. Lucia, QLD, Brisbane, 4072 Australia; 2 Australian Centre for Ecogenomics, The University of Queensland, St. Lucia, QLD, Brisbane, 4072 Australia; 3 Center for Integrated Protein Science Munich at the TUM School of Life Sciences, Technische Universität München, 85354 Freising, Germany; 4 Department of Chemistry, Maynooth University, Maynooth, Co. Kildare Ireland

**Dear Editor**,

Antibiotic resistance has emerged as a major threat to global health; multi-drug resistant bacteria already kill more patients in the United States each year than HIV/AIDS, Parkinson’s disease, emphysema and homicide combined (Laxminarayan et al., 2013). Among the most effective bacterial resistance mechanisms are β-lactamases, a family of enzymes that are divided into four distinct classes. Classes A, C and D (serine-β-lactamases, SBLs) use a catalytic site serine residue to initiate inactivation of the antibiotic, while Class B (metallo-β-lactamases, MBLs) relies on a Zn^2+^-activated hydroxide (Walsh et al., 2005; Bush and Jacoby, 2010; Mitic et al., 2014; Lisa et al., 2017). Clinically relevant inhibitors of Class C and D SBLs are available and in use (e.g., clavulanic acid (CA), Drawz et al., 2010), but for MBLs the search for such inhibitors has remained challenging (McGeary et al., 2017).

MBLs are divided into three subgroups, i.e. B1, B2 and B3 (Bush and Jacoby, 2010). Enzymes of the B1 subgroup constitute the majority of MBLs associated with antibiotic resistance (Khan et al., 2017). Fewer B2-type MBLs are currently known; they are phylogenetically related to B1 MBLs but are characterized by a preference for “last line” carbapenem substrates (Sun et al., 2016). While B3-type MBLs share low sequence similarity to B1 and B2 enzymes (< 20% amino acid (aa) identity), they have a substrate range similar to that of B1 MBLs (Selleck et al., 2016; Lee et al., 2019). MBLs contain catalytic centres that can accommodate two closely spaced Zn^2+^ ions bound in the α and β sites with similar yet distinct sequence motifs (B1: His116, His118, His196 and Asp120, Cys221, His263 (i.e., HHH/DCH) for the α and β sites, respectively; B2: NHH/DCH; B3: HHH/DHH).

For B3-type MBLs two variations of the canonical active site motif have been observed, **Q**HH/DHH in GOB-1/18 from the opportunistic pathogen *Elizabethkingia meningoseptica* and H**R**H/D**QK** in SPR-1 from *Serratia proteamaculans* (variations shown in bold) (Vella et al., 2013; Moran-Barrio et al., 2016). The discovery of atypical active sites in B3-type MBLs may have important implications for the design of clinically useful MBL inhibitors. We thus probed the evolutionary history and diversity of B3-type MBLs by searching for homologs in the release 02-RS83 of the Genome Taxonomy Database (Parks et al., 2018) comprising 111,330 quality-filtered bacterial and archaeal genomes. A total of 1,449 B3 MBL proteins were identified in 1,383 genomes (representing 1.2% of all analyzed genomes), of which 1,150 have the characteristic B3 active site residues (HHH/DHH), 162 the QHH/DHH and 47 the HRH/DQK motifs. In addition, we also discovered 90 proteins with another single aa variation in the α-site (EHH/DHH). Phylogenetic inference of a representative subset of 761 of these proteins indicates that each of the three motif variants originate from within the B3 radiation when using Class D SBLs as the outgroup (Fig. 1). We therefore propose to use the active site aa changes as a means of distinguishing the variants (i.e., B3-RQK, B3-Q, B3-E). B3-RQK appears to have only arisen once, likely because the ancestral change required at least four nucleotide (nt) substitutions to produce the three aa changes. By contrast, the B3-Q and B3-E variants have a single aa difference in position 116 requiring only one and two nt changes, respectively. The B3-Q variant appears to have arisen on at least six independent occasions and reverted back to the B3 motif on at least three occasions as a result of the need for only one nt change.

**Figure 1:**
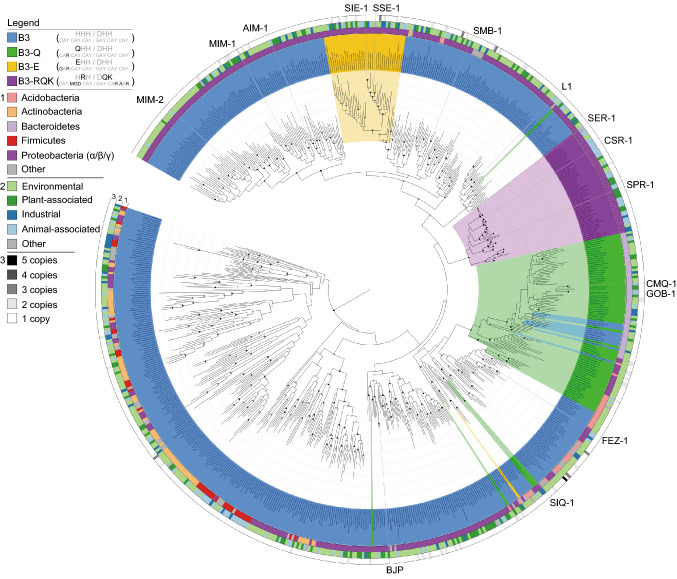
**Maximum likelihood tree of MBLs belonging to subgroup B3, highlighting three active site variants.** The tree was inferred from 688 dereplicated B3 MBLs identified in 1,383 bacterial genomes screened from a total of 111,330 bacterial and archaeal genomes. Bootstrap support for the interior nodes is indicated by filled (black: > 90%, gray: > 80%) or open (> 70%) circles. Representatives of class D SBLs were used as an outgroup for the analysis (not shown). B3 active site variants are indicated by different colors according to the legend in the top left of the figure. The inner circle (1) represents the phylum-level affiliations of the B3-containing bacteria. The middle circle (2) represents the habitat. Source of the B3-containing bacteria, and the outer circle (3) represents B3 gene copy number in each genome

No archaeal genomes harbored B3-type MBLs, and the majority were found in just four bacterial phyla; the *Proteobacteria*, *Actinobacteria*, *Bacteroidetes* and *Firmicutes* (Figs. 1 and S1). While this reflects to some extent the current over-representation of these phyla in the genome database (Fig. S2), it also suggests that the host range of B3 MBLs is relatively restricted. Between two and five B3 genes were found in 57 genomes, with the most copies being present in an as-yet-uncultured member of the *Acidobacteria* (Table S1). Numerous instances of native B3 enzymes co-occurring with B3-E and B3-Q were identified, however, only one instance of a B3 and B3-RQK was found (in a member of the *Enterobacteriaceae*) possibly indicating functional incompatibility of these enzymes.

The phylogenetic analysis of the B3 MBL family indicates a large and diverse reservoir of bacterial species potentially able to degrade β-lactam antibiotics. Standard B3 MBLs are potent β-lactamases as shown by *in vitro* β-lactam antibiotic degradation assays and their ability to confer *ex vivo* resistance to *Escherichia coli* (Yong et al., 2012). The only characterized representatives of B3-RQK (SPR-1) (Vella et al., 2013) and B3-Q (GOB-1/18) (Moran-Barrio et al., 2016) have β-lactamase activity, but only GOB-1/18 has been shown to confer resistance to a host organism. We therefore investigated if the ability to confer resistance to a bacterial host is a universal property among B3 enzymes. Twelve genes representing each of the B3 active site motif groups were selected for expression of the corresponding mature proteins in *E*. *coli*. Their ability to confer resistance against substrates that represent the three major classes of β-lactam antibiotics was assessed using disc tests (Tables S2 and S3). All MBLs, with the exception of B3-RQK enzymes, conferred resistance to at least one antibiotic of each class, and based on their aggregate resistance scores the subgroups can be ranked from most to least resistant as follows: B3 > B3-E > B3-Q. The B3-RQK enzyme SPR-1 was only catalytically active when it was truncated (by 49 aa) at the N-terminus (Vella et al., 2013). Indeed, the B3-RQK proteins SPR-1, CSR-1 and SER-1 could only confer resistance in truncated form (Table S2). Unlike SBLs, MBLs are largely resistant to CA (Fig. S3) confounding efforts to inhibit their activity against antibiotics (Walsh et al., 2005). Indeed, each of the B3, B3-E and B3-Q enzymes in our study were resistant. However, the truncated versions of the B3-RQK enzymes are sensitive to CA (Tables S2 and S3, Fig. S3); CSR-1_trunc_, SPR-1_trunc_ and SER-1_trunc_ were inhibited by CA with *K*_*i*_ values ranging from 200 μmol/L to 350 μmol/L, comparable to *K*_*i*_ values reported for Class D SBLs (20 to 200 μmol/L) (Drawz et al., 2010).

We determined the crystal structures of the mature CSR-1 protein and CSR-1_trunc_, and compared them to structures of B3 and B3-Q MBLs (Fig. S4). Only in the case of the mature CSR-1 structure is the N-terminal loop located above the active site, likely blocking substrate access. Removal of the N-terminus exposes the catalytic core, thus promoting activity. In all known MBLs the cavity of the catalytic centre can accommodate up to two Zn^2+^ ions (Mitic et al., 2014). However, no metal ions were observed in the crystal structures of CSR-1 and CSR-1_trunc_ (Fig. S4), suggesting reduced metal ion affinity. B3-RQK MBLs have three distinct aa variations in the active site motif, including two in the β site (Fig. 1). To probe the role of these residues single, double and triple mutants of CSR-1_trunc_ (i.e., CSR-1_trunc,sm_, CSR-1_trunc,dm_ and CSR-1_trunc,tm_) were generated to create variants where either the α, the β or both metal binding sites are identical to the canonical B3 MBL motif. These mutations had no significant effect on the overall structures, however, in CSR-1_trunc,sm_ electron density indicates the presence of one bound Zn^2+^ in the α-site, while in the double and triple mutants both the α- and β-sites are occupied by Zn^2+^ ions (Fig. 2A). The qualitative observation that the three mutations significantly enhance metal binding was confirmed using isothermal titration calorimetry (ITC; Fig. S5, Table S4).

**Figure 2:**
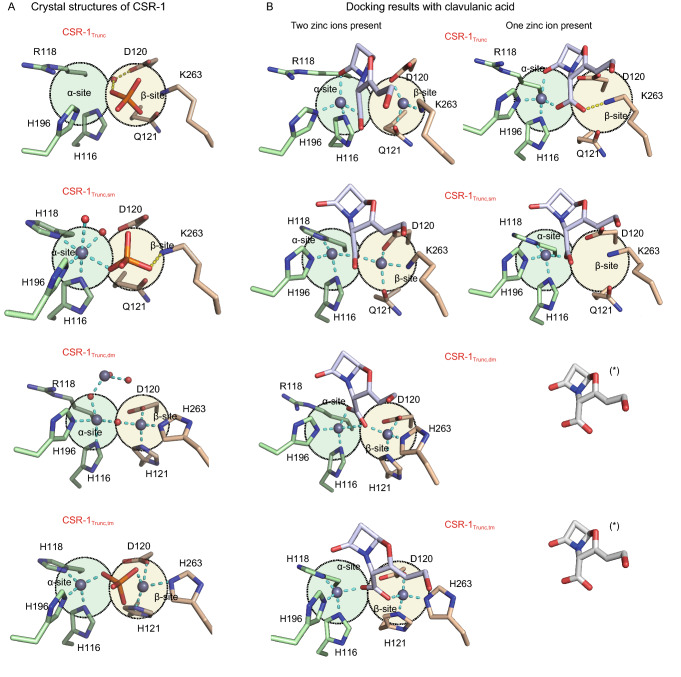
**Structural analysis of CSR-1 variants and their interaction with the inhibitor clavulanic acid. (A)** Crystal structures of the active sites of the B3-RQK enzyme CSR-1 and its mutants highlighting the α- (green circle) and β- (yellow circle) metal binding sites. (B) Predicted docking of CA to CSR variants in the presence of one or two Zn^2+^ metal ions. The predicted binding of the bi-metallic forms of CSR-1 and its mutants are unlikely to represent the inhibited form of this enzyme because there is no difference in the four poses. In the presence of one metal ion, however, CA (*) is predicted to only form a stable enzyme-inhibitor complex with CSR-1 and its single mutant, consistent with the experimental inhibition data (Table S5)

The increasing Zn^2+^ binding capacity of CSR-1_trunc_ as a function of introduced mutations was paralleled by enhanced *ex vivo* and *in vitro* activities, especially for the double and triple mutants; importantly, these two variants are also resistant to CA (Tables S5 and S6). Thus, the variations in the β-site of B3-RQK MBLs are responsible for the loss of catalytic activity and sensitivity to CA. *In silico* docking calculations with the bimetallic form of the enzyme predicted that the carboxylate group of CA binds to both metal ions and forms hydrogen bonds with Ser214, Asn254 and Arg257 in CSR-1_trunc_ and the three mutants (Figs. 2B and S6). When the weaker bound metal was removed (from the β site; Table S4), a stable enzyme-inhibitor complex was only formed in CSR-1_trunc_ and CSR-1_trunc,sm_, indicating that CA inhibits B3-RQK MBLs by displacing Zn^2+^ from the low affinity β site. Hydrogen bonding to Lys263 provides additional stabilization of the B3-RQK-CA (Fig. 2B).

In conclusion, despite being phylogenetically unrelated to their counterparts from the B1 and B2 subgroups, B3 MBLs have a similar active site motif, with similar catalytic properties (Lisa et al., 2017). Indeed, B3 MBLs enzymes are as efficient as B1 and B2 MBLs in inactivating a broad range of β-lactam antibiotics (Selleck et al., 2016), and are generally not inhibited by CA (Fig. S3; Table S2). Through a broad phylogenetic analysis of B3 MBLs detected in the rapidly expanding microbial genome database (Parks et al., 2018), we identified members with four distinct active site variations in a wide range of hosts and habitats (Figs. 1 and S1). B3-RQK is remarkable for its weak metal binding, its aa substitutions in the β-site, its occluded active site, reduced activity and sensitivity to CA (Tables S2–6, Figs. 2, S3 and S4). Mutation of the B3-RQK active site to restore the native B3 motif resulted in increased metal affinity, catalytic activity and resistance to CA (Tables S4–6). This can be primarily attributed to the restoration of the canonical histidine in position 263 of the β-site, and raises the possibility of modifying CA to effectively bind in the presence of His263, thereby increasing the therapeutic range of this widely used antibiotic resistance drug.

## Footnotes

This research was supported by Project Grants from the NH&MRC (APP1084778) and Australian Research Council (DP150104358), a Future Fellowship (FT120100694) awarded to GS, and a Laureate Fellowship (FL150100038) awarded to PH. NM was supported by a Science Foundation Ireland—President of Ireland Young Researcher Award (SFI-PIYRA). IA and OM were supported by the Deutsche Forschungsgemeinschaft (SFB 749, project C08; and CIPSM).

MMP, DWW, NM, RPM and GS devised the study. DWW and PH performed the phylogenetic analysis. MMP, NM, LW and LWG performed the experimental work. OM and IA performed molecular dynamics computations. MMP, DWW, PH and GS wrote the manuscript, and all authors contributed edits to the final version.

Marcelo Monteiro Pedroso, David W. Waite, Okke Melse, Liam Wilson, Nataša Mitić, Ross P. McGeary, Iris Antes, Luke W. Guddat, Philip Hugenholtz and Gerhard Schenk declare that they have no conflict of interest. This article does not contain any studies with human or animal subjects performed by the any of the authors.

## Electronic supplementary material

Below is the link to the electronic supplementary material.

Supplementary file1 (PDF 9423 kb)
