# Supplementary material for: Broad spectrum antibiotic-degrading metallo-β-lactamases are phylogenetically diverse
Source: Protein Cell. 2020 Jun 15;11(8):613–7. doi: 10.1007/s13238-020-00736-4 (PMC7381538; doi:10.1007/s13238-020-00736-4)
Supplement: Supplementary file 1 — Supplementary file1 (PDF 9423 kb) [file 13238_2020_736_MOESM1_ESM.pdf]

Supplementary Material

Broad spectrum antibiotic-degrading metallo-β-lactamases are phylogenetically diverse.

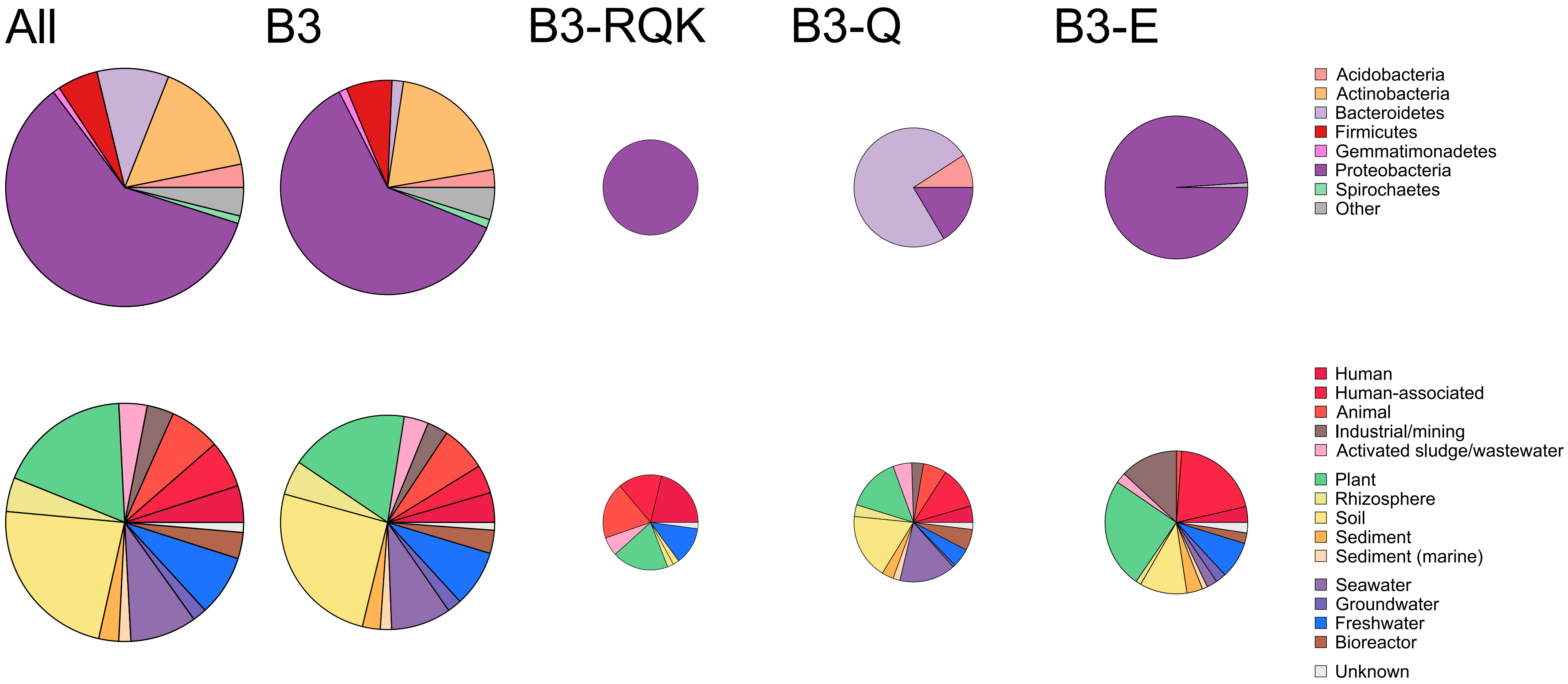

Fig. S1| Bacterial host affiliation and environmental distribution of B3 enzymes. Pie charts are scaled by the number of component protein sequences in each enzyme category. Host affiliation is shown for the top seven phyla and environments are categorised according to the legend to the right of the figure. Overlaying the B3 MBL tree with the host phylogeny (ring 1 in **Fig. 1**) indicates extensive lateral transfer of the MBL genes, most conspicuously at the phylum level. Using Class D SBLs as an outgroup suggests that B3 MBLs originated in the *Actinobacteria* and from there distributed to other phyla via lateral gene transfer. The B3-RQK and B3-E variants were found exclusively in the *Proteobacteria* and B3-Q were mostly identified in the *Bacteroidetes*. Localisation of the B3-RQK genes in the family *Enterobacteriaceae* and genus *Acinetobacter* is particularly striking, suggesting that these changes to the active site are relatively recent and lateral transfer has been limited thus far. A majority of habitats linked to the identified sequences are associated with humans (typically hospital or farming environments) or soil and rhizosphere samples (outer ring in **Fig. 1**). Previously studied, clinically relevant MBLs were not clustered together as a single monophyletic unit, but rather interspersed throughout the tree. This finding suggests that clinical MBLs have entered into the hospital environment through multiple, independent incursion events and are not the result of a single human-associated MBL undergoing diversification. Notably, an increasing proportion of B3-like MBLs were identified in water-borne bacteria in the deeper branching regions of the tree (closer to the Class D outgroup). Two interpretations of this observation are possible. Firstly, the higher proportion of B3-like MBLs in aquatic bacteria may reflect the result of human activity, whereby human-associated MBLs are mobilised into the water column. Alternatively and more likely, this observation may simply be the result of aquatic and marine environments as previously underestimated reservoirs of B3 MBLs.

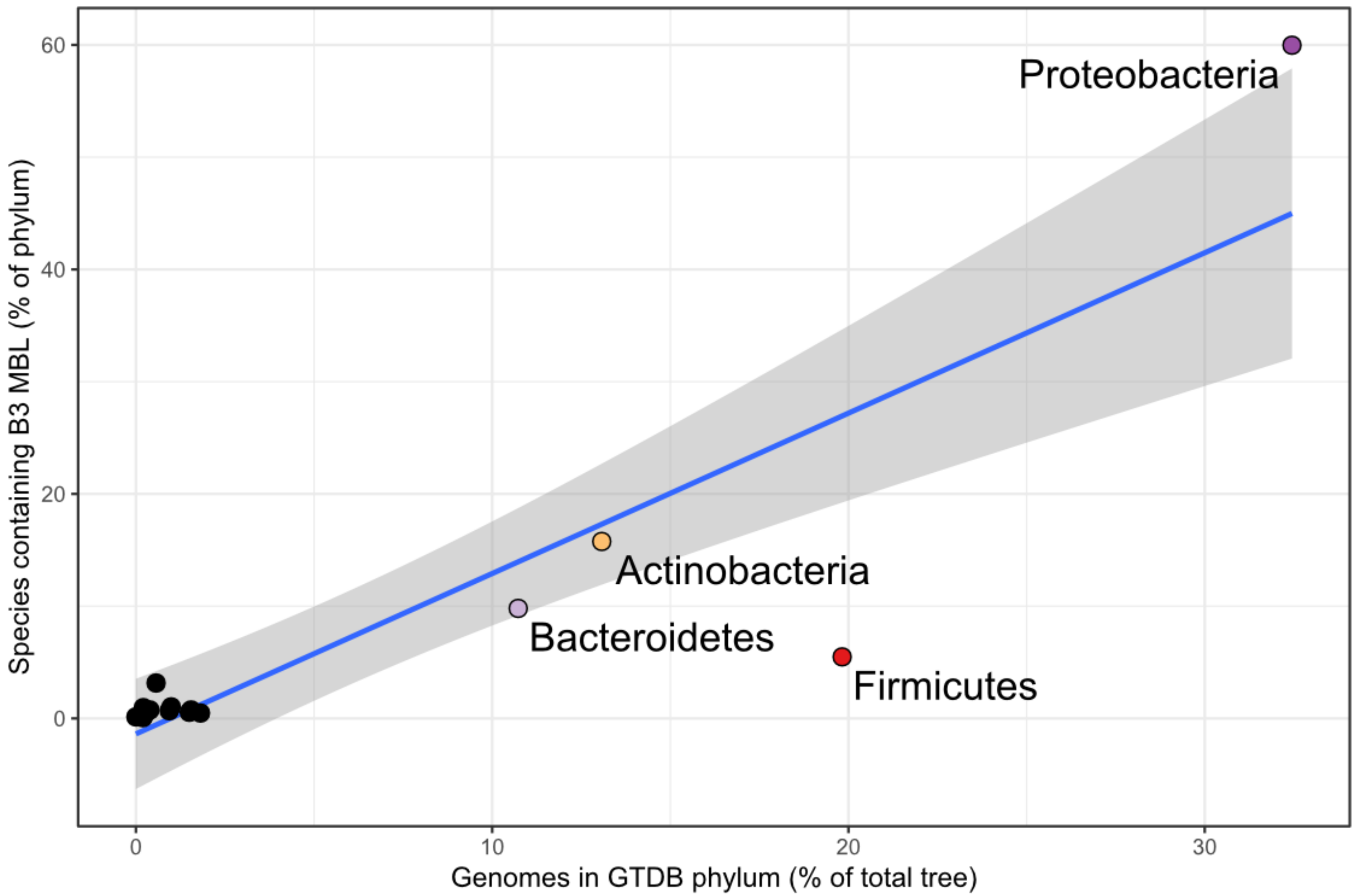

Fig. S2| Scatter plot of percentage total genomes vs percentage of discovered B3 MBLs in those genomes according to phylum affiliation. Dominant phyla in the genome database have the highest percentage of B3 MBLs, however, this correlation is not exact as *Proteobacteria* are relatively over-represented and *Firmicutes* relatively under-represented possibly reflecting greater activity of MBLs on Gram negative than Gram positive bacteria.

Table S1| Number of copies found in the different species.

Genome	Genome (NCBI)	Tip label	B3	B3-E	B3-Q	B3-RQK	Total
GB_GCA_001767525.1	GCA_001767525.1	Acidobacteria bacterium RIFCSPLOWO2_12_FULL_68_19	5	0	0	0	5
GB_GCA_001767195.1	GCA_001767195.1	Acidobacteria bacterium RIFCSPLOWO2_02_FULL_67_21	3	0	0	0	3
GB_GCA_001898925.1	GCA_001898925.1	Novosphingobium sp. 63-713	1	2	0	0	3
GB_GCA_001919455.1	GCA_001919455.1	Acidobacteria bacterium 13_1_40CM_2_56_11	3	0	0	0	3
RS_GCF_000379645.1	GCF_000379645.1	Actinoplanes globisporus DSM 43857	3	0	0	0	3
RS_GCF_001485665.1	GCF_001485665.1	Janthinobacterium sp. CG23_2	3	0	0	0	3
RS_GCF_001865675.1	GCF_001865675.1	Janthinobacterium sp. 1_2014MBL_MicDiv	2	1	0	0	3
GB_GCA_000974665.1	GCA_000961635.1	alpha proteobacterium U9-1i	2	0	0	0	2
GB_GCA_001464575.1	GCA_001464575.1	Acidobacteria bacterium Ga0077553	1	0	1	0	2
GB_GCA_001618865.1	GCA_001618865.1	Acidobacteria bacterium DSM 100886	0	1	1	0	2
GB_GCA_001899605.1	GCA_001899605.1	Sphingobacteriales bacterium 40-81	1	0	1	0	2
GB_GCA_001899685.1	GCA_001899685.1	Sphingobacteriales bacterium 44-15	1	0	1	0	2
GB_GCA_900103265.1	GCA_900103265.1	Sphingomonas sp. NFR15	1	1	0	0	2
GB_GCA_900114495.1	GCA_900114495.1	Dyella sp. OK004	1	1	0	0	2
RS_GCF_000014905.1	GCF_000014905.1	Candidatus Solibacter usitatus Ellin6076	1	0	1	0	2
RS_GCF_000017265.1	GCF_000017265.1	Phenylobacterium zucineum HLK1	2	0	0	0	2
RS_GCF_000204015.1	GCF_000204015.1	Asticcacaulis biprosthecum C19	2	0	0	0	2
RS_GCF_000242815.1	GCF_000242815.1	Janthinobacterium lividum PAMC 25724	1	1	0	0	2
RS_GCF_000253255.1	GCF_000253255.1	MIM-1	2	0	0	0	2
RS_GCF_000281975.1	GCF_000281975.1	Novosphingobium sp. AP12	1	1	0	0	2
RS_GCF_000374905.1	GCF_000374905.1	Arthrobacter sp. 162MFSha1.1	2	0	0	0	2
RS_GCF_000381565.1	GCF_000381565.1	Oxalobacteraceae bacterium AB_14	2	0	0	0	2
RS_GCF_000382885.1	GCF_000382885.1	Sphingobium japonicum BiD32	2	0	0	0	2
RS_GCF_000447205.1	GCF_000447205.1	Sphingobium ummariense RL-3	1	1	0	0	2
RS_GCF_000473995.1	GCF_000473995.1	Clostridium saccharobutylicum DSM 13864	2	0	0	0	2

RS_GCF_000632105.1	GCF_000632105.1	Novosphingobium resinovorum	2	0	0	0	2
RS_GCF_000744465.1	GCF_000744465.1	Caulobacter henricii	2	0	0	0	2
RS_GCF_000763945.1	GCF_000763945.1	Sphingopyxis sp. MWB1	1	1	0	0	2
RS_GCF_000813185.1	GCF_000813185.1	Novosphingobium sp. MBES04	1	1	0	0	2
RS_GCF_000829715.2	GCF_000829715.2	Streptomyces sp. NBRC 110027	2	0	0	0	2
RS_GCF_001298105.1	GCF_001298105.1	Novosphingobium sp. ST904	1	1	0	0	2
RS_GCF_001421325.1	GCF_001421325.1	Novosphingobium sp. Leaf2	1	1	0	0	2
RS_GCF_001426905.1	GCF_001426905.1	Caulobacter sp. Root1455	2	0	0	0	2
RS_GCF_001427665.1	GCF_001427665.1	Caulobacter sp. Root655	1	1	0	0	2
RS_GCF_001449105.1	GCF_001449105.1	Caulobacter vibrioides	1	1	0	0	2
RS_GCF_001591025.1	GCF_001591025.1	Sphingomonas soli NBRC 100801	2	0	0	0	2
RS_GCF_001591045.1	GCF_001591045.1	Sphingopyxis granuli NBRC 100800	2	0	0	0	2
RS_GCF_001598415.1	GCF_001598415.1	Sphingomonas mali NBRC 15500	1	1	0	0	2
RS_GCF_001717955.1	GCF_001717955.1	Sphingomonas panacis	1	1	0	0	2
RS_GCF_002001165.1	GCF_002001165.1	Rhodanobacter sp. C05	2	0	0	0	2
RS_GCF_002007115.1	GCF_002007115.1	Massilia sp. KIM	2	0	0	0	2
RS_GCF_900011245.1	GCF_900011245.1	Bradyrhizobium sp.	2	0	0	0	2
RS_GCF_900156165.1	GCF_900156165.1	Pseudacidovorax sp. RU35E	2	0	0	0	2
RS_GCF_900177785.1	GCF_900177785.1	Cedecea sp. NFIX57	1	0	0	1	2
U_66028	SAMN08018157	Gammaproteobacteria bacterium UBA8143	1	0	1	0	2
U_66329	GCA_002311835.1	Proteobacteria bacterium UBA1148	0	0	2	0	2
U_67505	SAMN08019550	Gammaproteobacteria bacterium UBA8658	1	0	1	0	2
U_67723	GCA_002336595.1	Gammaproteobacteria bacterium UBA1979	1	0	1	0	2
U_68064	GCA_002328835.1	Acidobacteria bacterium UBA2161	2	0	0	0	2
U_69330	GCA_002347025.1	Acidobacteria bacterium UBA2990	2	0	0	0	2
U_69335	GCA_002346545.1	Acidobacteria bacterium UBA2994	1	0	1	0	2
U_72084	GCA_002387245.1	Brevundimonas sp. UBA4553	1	1	0	0	2

U_74706	SAMN08018207	Janthinobacterium bacterium UBA11349	2	0	0	0	2
U_74816	GCA_002428565.1	Anaerolineales bacterium UBA6092	2	0	0	0	2
U_75494	GCA_002434915.1	Proteobacteria bacterium UBA6522	0	0	2	0	2
U_77156	GCA_002477465.1	Gammaproteobacteria bacterium UBA7449	1	0	1	0	2
U_77191	GCA_002478575.1	Sphingomonadales bacterium UBA7473	1	1	0	0	2

Table S2| *In vitro* kinetic data and *ex vivo* plate tests of selected B3 metallo-β-lactamases. As a positive control, genes of known B3 MBLs (L1, FEZ-1, AIM-1, MIM-1 and MIM-2) were included in the analysis.

Class	Enzyme	Host species	Pathogenicity	Habitat	*E. coli* plate test (*in vitro* kinetics, s^{-1})[a]									Aggregate Resistance Score[c]	Clavulanic acid	UniProt accession	PDB	Reference
					Penams			Cephalosporins			Carbapenems							
					AMP	CAR	PEN	NIC	CXM	CEF	MEM	BPM	IPM					
B3	L1	*Stenotrophomonas maltophilia*	Y	animal	R (580)	R	R (410)	R (40)	R (53)	R (65)	R	R (64)	R (48)	9	R	BLA1_STEMA	1SML	Ullah *et al.*, 1998
	FEZ-1	*Legionella gormanii*	N	soil			(70)	(100)	(320)	(300)	(45)	(70)	(200)		R	Q9K578_9GAMM	1K07	Garcia-Saez *et al., 2003*
	AIM-1	*Pseudomonas aeruginosa*	Y	animal	R (24)	R	R (110)	R (22)	R (35)	R	S (41)	R	R (410)	8	R	B5DCA0_PSEAI	4AWY	Leiros *et al., 2012*
	MIM-1	*Novosphingobium pentaromativorans*	N	aquatic	R (1195)	R	R (237)	R (18)	R (60)	S (1.8)	S (54)	R (143)	R (97)	7	R	NSU_3853	6AUF	Selleck *et al., 2020*
	MIM-2	*Simiduia agarivorans*	N	aquatic	R (701)	R	R (201)	R (7)	R (98)	r (40)	S (1.6)	R (5.3)	R (7)	7.5	R	M5M_14960	6MFI	Selleck *et al., 2020*
B3-RQK[b,d]	SPR-1$_{Trunc}$	*Serratia proteamaculans*	N	animal/ plant	R (259)	S	r (176)	R	r	S (17)	S	r	R	5	S	Spro_3259		Vella *et al., 2013*
	CSR-1$_{Trunc}$	*Cronobacter sakasakii*	Y	foodborne	R (11)	S (1)	r (17)	R (2)	r (1)	S (0.7)	S (6)	R (9)	R (8)	5	S	CSK29544_03680	6DN4	
	SER-1$_{Trunc}$	*Salmonella enterica*	Y	animal	R (189)	S (0.7)	r (2)	R (25)	R (7)	S	S (1.5)	R (27)	r (5)	5	S	C4855_18475		
B3-Q	GOB-1	*Elizabethkingia meningoseptica*	Y	water/ soil	R	r	R (540)	R (20)	R	r (24)	r (100)	S	R (77)	6.5	R	blaGOB-1	5K0W	Moran-Barrio *et al., 2016*
	CMQ-1	*Chryseobacterium molle*	N	animal/ plant	R	S	r	R	R	S	S	S	R	4.5	R	SAMN05444371_2870		
	SIQ-1	*Sphingomonas indica*	N	soil	R	R	R	R	R	R	S	S	R	7	R	WP_085219180		
B3-E	SIE-1	*Sphingobium indicum*	N	soil	R	R	R	R	R	S	S	S	R	6	R	SIDU_11385		
	SSE-1	*Sphingopyxis sp.*	N	soil	R	R	R	R	R	r	S	S	R	6.5	R	SPPYR_1125		

a. All plate tests were performed in the present study and some *in vitro* kinetic data were obtained from published studies. b. The signal peptide was removed from all proteins (~25 aa). An additional 30 to 50 aa were removed from the N-terminus of the B3-RQK variants (*see text*); SPR-1 (49 aa), CSR-1 (32 aa), SER-1 (35 aa). c. Aggregate resistance scores of the MBLs were calculated as the sum of individual resistance scores for each of nine substrates. The scoring system was 1 for resistance (R; zone of inhibition <18 mm), 0.5 for marginal resistance (r; inhibition zone 18-26 mm) and 0 for sensitivity (S; inhibition zone ≥27 mm) based on zones observed around the negative control. [d] Previously called B4 (Vella et al., 2013).

Table S3| *Ex vivo* sensitivity disc test. MBLs were given aggregate scores (**Table S2**) against nine substrates; 0 for sensitive (S; ≥ 27 mm zone of inhibition), 0.5 for marginal resistance (26≥ r >18 mm), and 1 for resistant (17 ≥ R > 5 mm; zone of inhibition).

Class	Enzyme	Host species	Sensitivity Tests – results in millimetres (mm)									Clavulanic Acid Inhibition	
			Penams			Cephalosporins			Carbapenems				
			AMP	CAR	PEN	NIC	CXM	CEF	MEM	BPM	IPM	AML[a]	AMLC[b]
B3	L1	*Stenotrophomonas maltophilia*	5±1	15±5	14±7	11±2	22±7	12±7	22±3	20±1	10±7	5±7	5±5
	AIM-1	*Pseudomonas aeruginosa*	5.0±1	10±1	15±3	12±1	13±3	21±4	36±7	12±7	10±2	7±5	6±4
	MIM-1	*Novosphingobium pentaromativorans*	18±4	15±7	21±3	10±7	5±7	34±7	40±6	15±5	12±5	5±3	5±2
	MIM-2	*Simiduia agarivorans*	18±2	10±4	7±4	19±3	5±7	24±2	42±5	12±3	12±7	8±4	7±3
B3-RQK	SPR-1_{Trunc}	*Serratia proteamaculans*	5±2	42±3	20±2	12±3	22±2	45±3	44±2	24±2	16±1	6±4	16±4
	CSR-1_{Trunc}	*Cronobacter sakasakii*	10±2	44±3	18±4	15±1	21±3	43±2	47±2	15±3	12±2	5±7	17±2
	SER-1_{Trunc}	*Salmonella enterica*	5±1	47±3	19±4	12±3	9±4	44±4	45±1	14±2	18±3	7±3	28±4
B3-Q	GOB-1	*Elizabethkingia meningoseptica*	8±4	19±2	5±2	6±4	8±2	18±3	21±4	47±3	5±4	5±4	5±1
	CMQ-1	*Chryseobacterium molle*	7±4	41±3	18±2	10±7	9±7	27±4	30±4	30±7	5±4	5±3	4±8
	SIQ-1	*Sphingomonas indica*	10±4	5±2	7±4	6±3	5±2	9±3	33±6	30±8	5±5	6±5	5±9
B3-E	SIE-1	*Sphingobium indicum*	15±2	10±6	7±4	5±5	5±2	43±7	40±3	35±4	5±1	9±4	9±2
	SSE-1	*Sphingopyxis sp.*	5±3	5±7	10±2	9±5	5±2	18±2	33±6	41±4	5±4	7±4	6±1

[a] Amoxycillin. [b] Amoxycillin and clavulanic acid

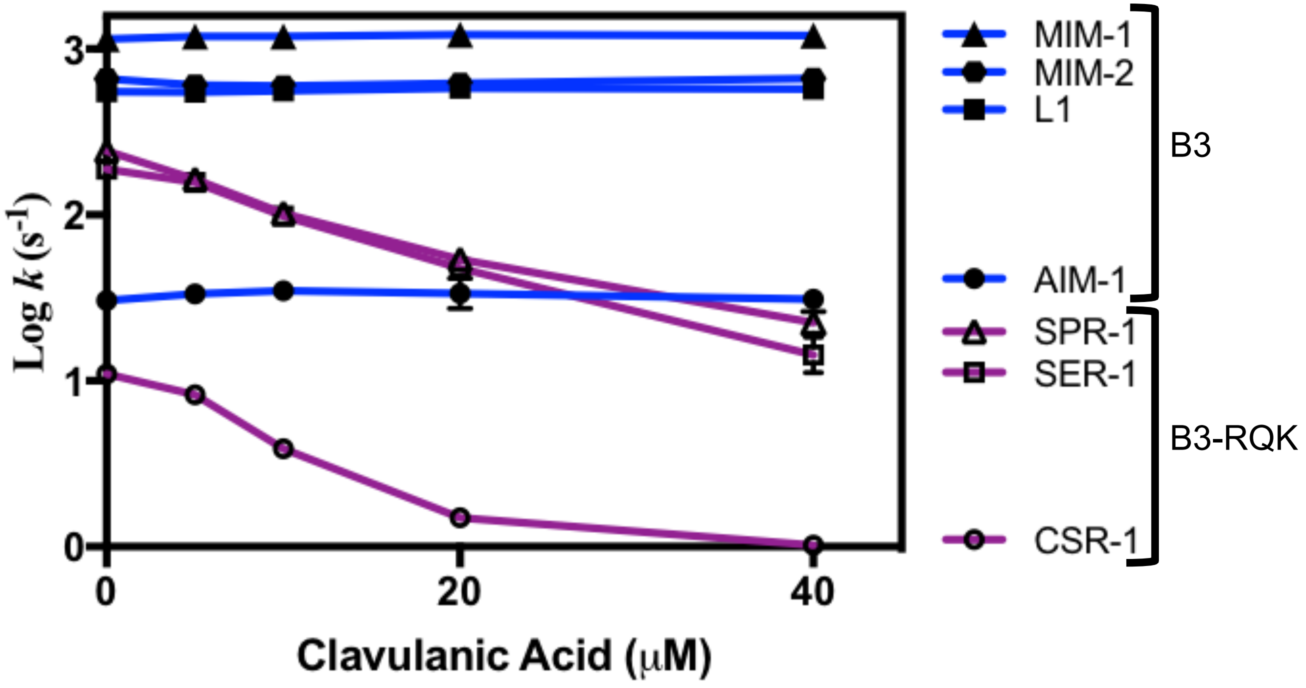

Fig. S3| Inhibition of B3-MBLs by clavulanic acid. The plot shows the effect of different concentrations of clavulanic acid *versus* the logarithm of k_{cat} values of selected B3-MBLs. The inhibitory effect, based on the constant K_i, is 350 µM for CSR-1$_{trunc}$, 201 µM for SPR-1$_{trunc}$ and 290 µM for SER-1$_{trunc}$, comparable to K_i values observed in SBLs (20 to 200 µM) (Drawz *et al.*, 2010), and in the only MBL for which a K_i for clavulanic acid (67 µM) has been reported (the B1-type MBL SPM-1; Murphy *et al.*, 2003).

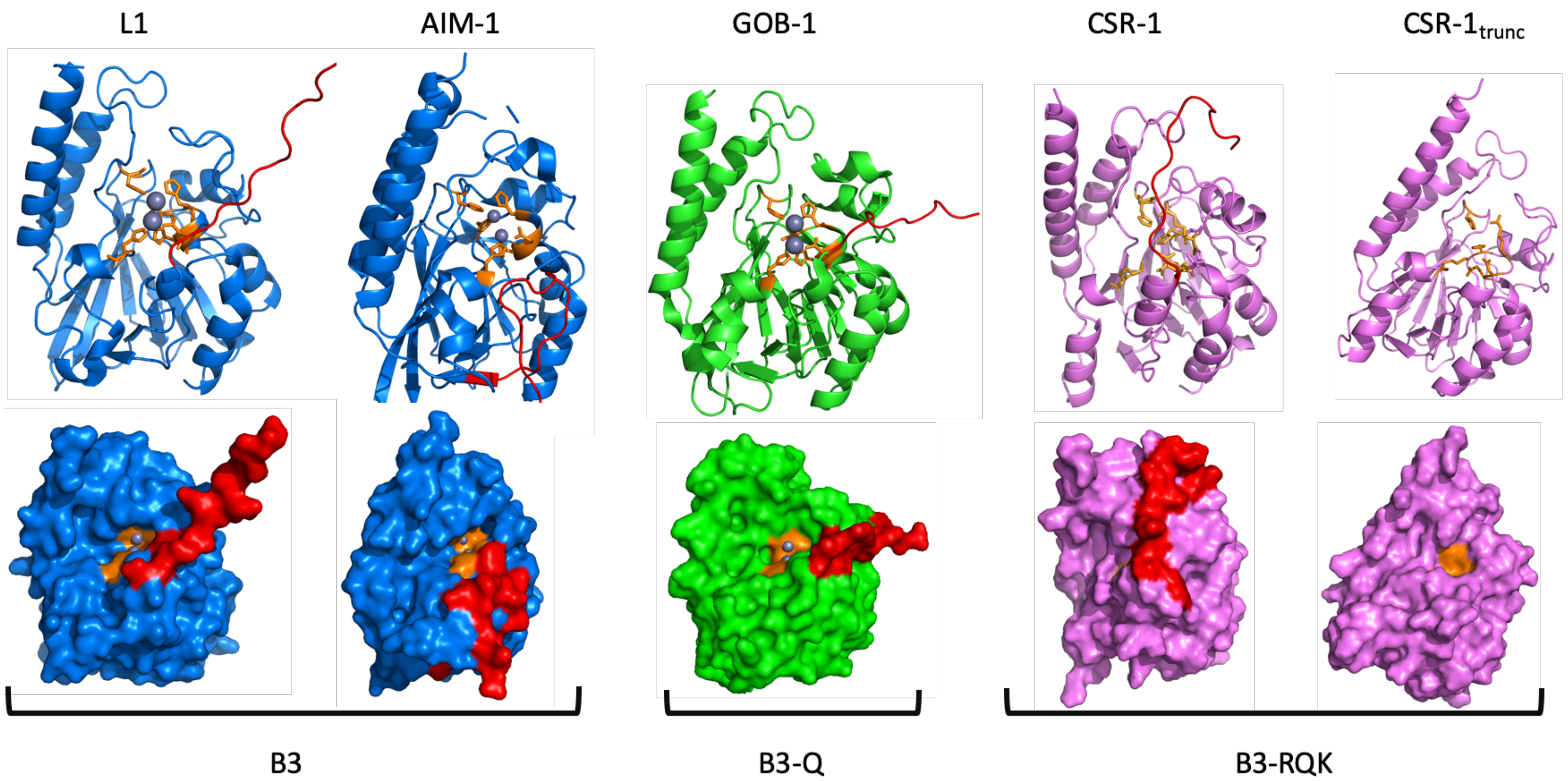

Fig. S4| Selected crystal structures of native B3-MBLs and two active site variants. The names of the specific enzymes are indicated at the top of the figure (highlighted in **Fig 1**), and the type of active site is noted at the base of the figure. The top panel shows cartoon representations of the overall structure of the selected enzymes, with active site ligands in orange, Zn^{2+} ions in grey, and the N-terminal loop in red. The bottom panel shows surface representations of the five structures highlighting occlusion of the active site by the N-terminus in CSR-1. A conspicuous difference between the four enzymes is the relative position of their N-termini. In L1, the N-terminus protrudes from the structure thereby making the active site accessible, whereas in AIM-1, a disulphide bridge between Cys32 and Cys66 locks its N-terminus in a position away from the catalytic centre (Leiros *et al.*, 2012; Hou *et al.*, 2017). Similar to L1, GOB-1/18 has a protruding N-terminus exposing the active site (Horsfall *et al.*, 2011; Moran-Barrio *et al.*, 2016).

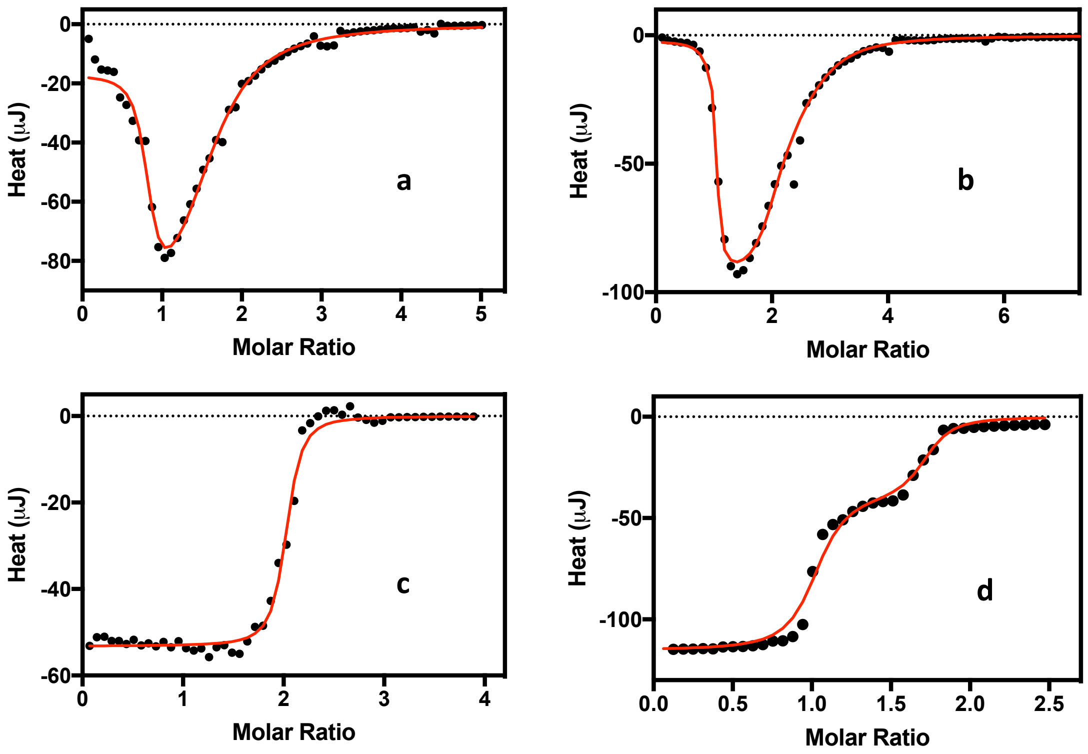

Fig. S5| Experimental ITC data collected for the variants of CSR-1 from the subgroup B3-RQK. Data for CSR-1$_{trunc}$ (a), CSR-1$_{trunc, sm}$ (b), CSR-1$_{trunc,dm}$ (c) and CSR-1$_{trunc,tm}$ (d) were fitted using a nonlinear algorithm to minimise χ^2 values using equations derived from equilibrium-binding models simulating either one or two independent binding sites.

Table S4| ITC parameters collected for CSR-1$_{trunc}$ and its mutants. CSR-1$_{trunc}$ binds two Zn^{2+} ions with moderate to weak affinities (αK_d ~2 µM and βK_d ~150 µM). Two Zn^{2+} ions also bind to CSR-1$_{trunc,sm}$, but the affinity of the α-site (αK_d ~0.1 µM) is significantly enhanced, while the β-site (βK_d ~180 µM) is unaffected by the mutation. In the double and triple mutants both the α and β metal binding sites have high affinity ($\alpha K_d = \beta K_d$ ~0.14 µM and αK_d ~0.06 µM and βK_d ~0.4 µM, respectively). For comparison, similar ITC measurements with AIM-1 result in an αK_d and βK_d of ~0.17 µM (Selleck *et al.*, 2016) indicating that the double and triple mutants of CSR-1 fully restore the characteristic ability of native B3 enzymes to bind two Zn^{2+} ions tightly.

	**CSR-1$_{trunc}$**	**CSR-1$_{trunc,sm}$**	**CSR-1$_{trunc,dm}$**	**CSR-1$_{trunc,tm}$**
Model[(a)]	Two Independent sites	Two Independent sites	One Independent Site	Two Independent sites
N_1	0.95±0.09	0.98±0.07	1.99±0.1	0.99±0.9
K_{d1} (µM)	2±0.7	0.1±0.07	0.14±0.07	0.06±0.02
N_2	0.87±0.06	1.2±0.06	-	0.83±0.1
K_{d2} (µM)	150±12	180±27	-	0.4±0.1

[(a)]Mathematical model applied to fit the experimental data. N is the stoichiometry of an interaction measured by ITC, and K_d is the dissociation constant.

Table S5. ***In vitro*** **kinetic data and** ***ex vivo*** **plate test of CSR-1 and its mutants.** The effect of the single mutation in the α-site (*i.e.* R118H) on the catalytic properties is modest; CSR-1$_{trunc,sm}$ displays a slightly enhanced catalytic activity towards most of the substrates tested (**Table S6**), but this positive effect is largely offset by reduced substrate binding. The effect of the double and triple mutations in CSR-1$_{trunc}$, however, is striking. A marked increase in catalytic efficiency was observed against most antibiotics accompanied by an increase in *ex vivo* resistance to both antibiotics (resistance score 8.5 vs 5 for CSR-1$_{trunc}$) and clavulanic acid.

CSR-1 enzyme	Length (aa)	Active site motif	*E. coli* plate test and *in vitro* kinetics (k_{cat} s^{-1})									Aggregate Resistance Score	Clavulanic acid
			Penams			Cephalosporins			Carbapenems				
			AMP	CAR	PEN	NIC	CXM	CEF	MEM	BPM	IPM		
trunc		H**R**H/D**QK**	R (11)	S (1)	r (17)	R (2)	r (1)	S (0.7)	S (6)	R (9)	R (8)	5	S
trunc,sm		HHH/D**QK**	R (23)	S (1.3)	r (12)	R (3)	r (1.7)	r (2)	r (12)	R (13)	R (10)	6	S
trunc,dm		H**R**H/DHH	R (89)	R (53)	R (100)	R (23)	R (60)	R (5)	R (152)	r (31)	R (27)	8.5	R
trunc,tm		HHH/DHH	R (170)	R (91)	R (78)	R (12)	R (110)	r (21)	R (251)	R (110)	R (183)	8.5	R
mature		H**R**H/D**QK**	S	S	S	S	S	S	S	S	S	0	nd
leader[b]		H**R**H/D**QK**	S	S	S	S	S	S	S	S	S	0	nd

Table S6| Steady-state kinetic parameters for CSR-1$_{trunc}$, CSR-1$_{trunc,sm}$, CSR-1$_{trunc,dm}$ and CSR-1$_{trunc,tm}$.

	k_{cat} (s^{-1})			
**Substrate**	**CSR-1$_{trunc}$**	**CSR-1$_{trunc,sm}$**	**CSR-1$_{trunc,dm}$**	**CSR-1$_{trunc,tm}$**
Ampicillin	11±2	23±8	89±7	170±9
Penicillin G	17±3	12±7	100±9	78±2
Carbenicillin	1±0.7	1.3±1	53±2	91±7
Cefuroxime	1±0.9	1.7±0.9	60±3	110±7
Cefoxitin	0.7±0.6	2±0.7	5±1	21±8
Nitrocefin	2±0.3	3±1	23±7	12±1
Imipenem	8±1	10±2	31±2	183±13
Meropenem	6±3	12±2	152±9	251±3
Biapenem	9±2	13±5	27±7	110±9

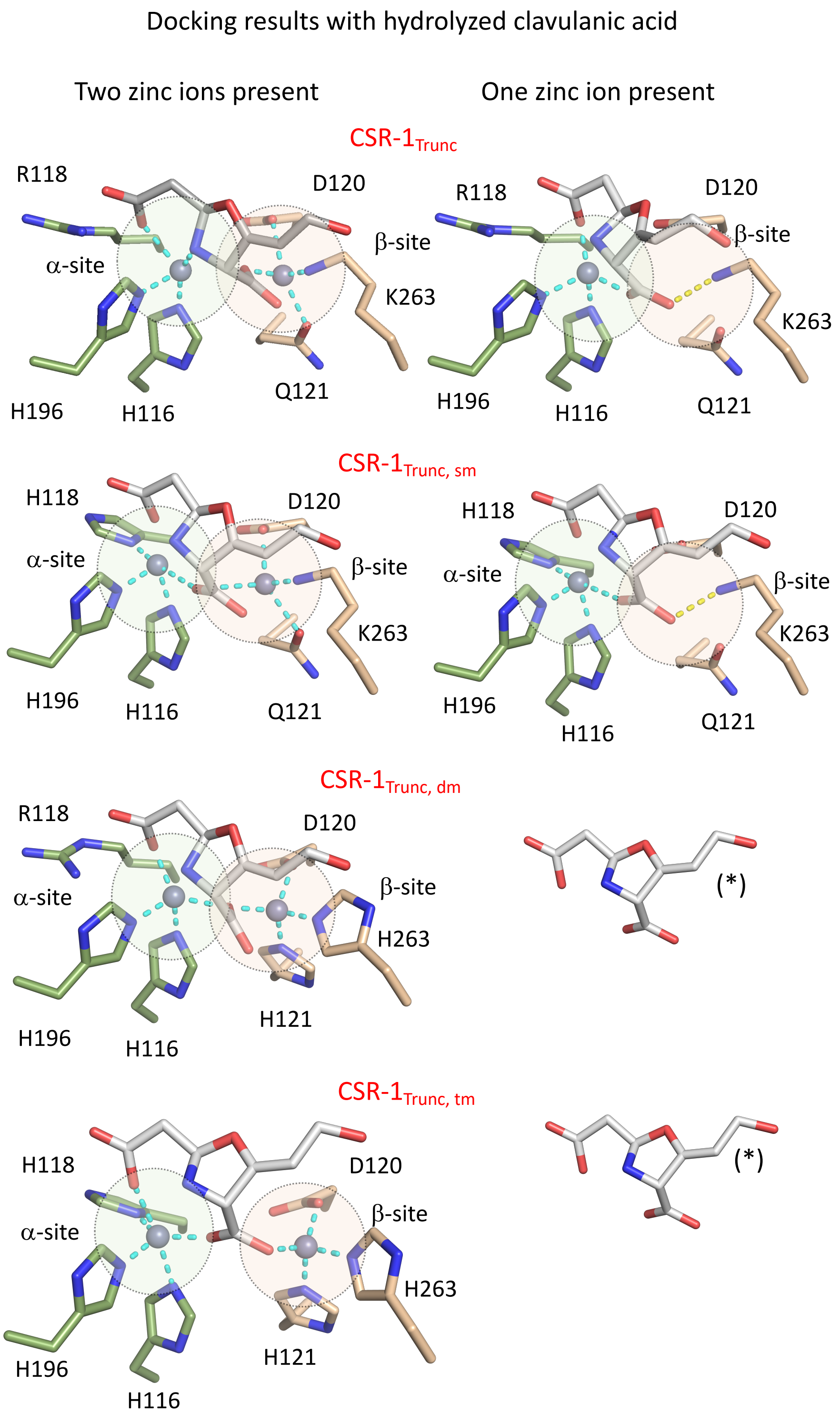

Fig. S6| Structural analysis of CSR-1 variants and their interaction with the hydrolyzed (open ring) inhibitor clavulanic acid. See **Fig. 2b** for experimental details. The mode of inhibitor binding is the same irrespective if clavulanic acid is intact or cleaved. The inhibitor acts by displacing the weaker bound Zn^{2+} in the β site of CSR-1$_{Trunc}$ and CSR-1$_{Trunc,sm}$.

Methods

Phylogenetic Analysis

Putative protein orthologues of the B3 family of MBLs were identified from the Genome Taxonomy Database using GeneTreeTK (version 0.0.11; https://github.com/dparks1134/GeneTreeTk). Sequences were manually curated, then aligned with MAFFT (Katoh and Standley, 2013). Columns representing six residues critical for metal ion binding were manually identified in the L1 alignment (His105, His107, Asp109, His110, His181, and His246), and proteins categorised according to their motif (B3: HHDHHH, B3-RQK HRDQHK, B3-Q: QHDHHH and B3-E EHDHH). For phylogenetic inference, sequences were dereplicated into clusters of proteins sharing at least 70% amino acid identity using usearch (v8.1) (Edgar, 2010). A total of 761 protein sequences (518 B3, 77 for B3-Q77, 35 for B3-E and 43 for B3-RQK), including an outgroup of 88 MBLs from Class D, were aligned using MAFFT and columns filtered using TrimAl (Katoh and Standley, 2013). Model parameters for phylogenetic inference were evaluated using ModelFinder (Kalyaanamoorthy *et al.*, 2017) in IQ-Tree (Nguyen et al., 2015), selecting the optimal model according to the Bayesian Information Criterion. Inference was then performed in IQ-Tree using the WAG+F+R10 model of amino acid substitution and 100 bootstrap re-samplings to assess node support. The resulting tree was visualised and annotated with genome metadata using the Interactive Tree of Life resource (Letunic and Bork, 2016).

Ex vivo whole cell plate assays

All genes listed in **Table S2**, with the exception of FEZ-1, were synthesised by General Biosystems. Genes were cloned into pET22b(+) and transformed into *E. coli* BL21(DE3) cells.

Transformed bacterial suspensions (0.5 McFarland units) were plated onto Mueller-Hinton (MH) agar. Antibiotic resistance tests were similarly performed with diffusion discs containing penicillin G, carbenicillin, cephalothin, nitrocefin, cefuroxime, meropenem, imipenem and biopenem. Plates were incubated at 35°C for 18 hours to allow bacterial growth, and zones of clearing around strips and/or discs were measured with a calliper against a dark background. Tests were conducted in quadruplicate, and the inhibition zone measurements averaged. *E. coli* cells with pET22b(+) plasmids lacking inserts were used as negative controls. Strip and disc tests were conducted according to the European Society of Clinical Microbiology and Infectious Diseases (EUCAST) standard for antimicrobial susceptibility testing.

Protein expression and purification

CSR-1, CSR-1_{trunc}, CSR-$1_{trunc,sm}$, CSR-$1_{trunc,dm}$ and CSR-$1_{trunc,tm}$ constructs were transformed into chemically competent *E. coli* LEMO BL21 (DE3) cells by heat shock. A single colony was inoculated into 20 mL Luria-Bertani medium supplemented with 50 µg/ml of kanamycin and 30 µg/ml of chloramphenicol, and the cultures were grown overnight at 37°C and then inoculated into Terrific Broth medium (1 L) and grown at 37°C until an optical density (OD_{600}) of 0.4-0.6 was reached. The temperature was then reduced to 18°C and liquid media incubated for an additional 48 hours. Subsequently, the cells were harvested by centrifugation at 4°C and resuspended in 20 mM HEPES pH 7.5 and 0.15 mM of $ZnCl_2$, protease inhibitor cocktail, 1 mg/mL lysozyme, 20 µg/mL of DNAse and $MgCl_2$ (5mM). Cells were disrupted by sonication and the supernatant was loaded into a 25 mL SP Sepharose FF column (GE Healthcare) equilibrated with 20 mM HEPES pH 7.5 and 0.15 mM of $ZnCl_2$. Expressed target proteins were eluted using a gradient of 1M NaCl in 20 column volumes.

Crystallization, X-ray diffraction data collection and refinement

Crystals of the expressed proteins were grown at 18°C by hanging-drop vapour diffusion using a 24-well pre-greased plate (Hampton Research) with drops containing 1 μL of the protein solution and 1 μL of precipitant buffer comprising 0.49 M of $NaHSO_4$, 0.91 M of $KHSO_4$ and 50 % w/v PEG 400. Diamond like crystals were observed after five days of incubation. Harvested crystals were cryoprotected in precipitant buffer containing 20% glycerol. Diffraction data were collected from cryoprotected crystals on beamline MX-2 at the Australian Synchrotron (Melbourne) using BLU-ICE (McPhillips *et al.*, 2002). Diffraction data were integrated, scaled and merged using the software HKL-2000 (Otwinowski and Minor, 1997). Refinement and model building were carried out using PHENIX 1.8.4 (Adams *et al.*, 2010) and COOT 0.7 (Emsley *et al.*, 2010), respectively. The CSR-1 and CSR-1_{trunc} structures were determined using the previously published coordinates for L1 (PDB 1SML) (Leiros *et al.*, 2012). The mutants CSR-$1_{trunc,sm}$, CSR-$1_{trunc,dm}$ and CSR-$1_{trunc,tm}$ were then solved by molecular replacement using the CSR-1 structure as a search model. All atoms were subsequently refined with anisotropic B-factors; most hydrogen atoms were fitted as riding models. Relevant crystallographic data and refinement statistics are summarised in **Table S7.**

Enzyme Kinetics

All kinetic measurements were performed on a Cary 60 Bio Varian UV-Vis spectrophotometer. In a standard assay, reactions were initiated by the addition of the enzyme and monitored for 60 s at 25°C. The Michaelis-Menten (Segel, 1993) parameters were determined by nonlinear fitting using Graphpad Prism 7. The hydrolysis of ampicillin, penicillin G, carbenicillin, nitrocefin, cefoxitin, cefuroxime, meropenem, biapenem, imipenem and nitrocefin were monitored in 50 mM TrisHCl (pH 8.5) by detecting the hydrolysis of the substrate. Inhibition measurements were obtained by

measuring the activity of the enzyme with ampicillin and analysing the effect of the increase in clavulanic acid concentration on the enzyme activity. The data were calculated using the mixed-inhibition equation (Segel, 1993).

Metal binding studies

The metal-free apoforms of the CSR-1 enzyme and its mutants were obtained by incubating approximately 3 mg of protein in a 3 mL solution containing 10 mM of EDTA in 20 mM HEPES buffer (pH 7.0 at 4°C). After 24 hours, protein samples were separated from the chelating solution using an Econo-Pac 10DG gel filtration column equilibrated with fresh HEPES buffer treated with chelex resin to remove any residual metal ions. Atomic absorption spectroscopy (AAS) was used to confirm the absence of metal ions in the final protein solutions. Zn^{2+} ions were then titrated back into the protein solutions and the heat release measured by isothermal titration calorimetry (ITC) at 25°C using a Nano ITC from TA instruments. The concentration of the metal ion solution was verified against standardised EDTA solutions and the results were compared against AAS data. At least four sets of data were collected per protein and the average calculated. The ITC data were fitted using TRIOS v4.4.0 software from TA instruments. The software uses a nonlinear algorithm to minimise χ^2 values to fit the experimental data to equations derived from equilibrium-binding models simulating either one or two independent binding sites (**Table S4**) (Pedroso *et al.*, 2014; Pedroso *et al.*, 2017).

Molecular docking and QM/MM calculations

For the theoretical studies, the missing Zn^{2+} ions in CSR-1_{trunc} and CSR-$1_{trunc,sm}$ were manually placed in the binding site using CSR-$1_{trunc,tm}$ as a template. All water molecules were removed, as

well as the artefactual third Zn^{2+} in CSR-$1_{trunc,dm}$ (**Fig. 2a**). Proteins were protonated using the program *tleap* in the AmberTools16 software package (Case *et al.*, 2016). Metal-ligating histidine residues were protonated at the nitrogen not involved in metal coordination. Lys263 in the β-site was considered in its neutral form such that it can ligate a Zn^{2+} ion. Molecular docking of clavulanic acid (both in intact and hydrolysed form) was performed with FlexX (Rarey *et al.*, 1996) within the LeadIT platform version 2.3.2 (https://www.biosolveit.de/LeadIT/). All residues within 10 Å of the geometric centre of the two metal ions were considered as binding site residues. The 'Enthalpy and Entropy' (*i.e.* Hybrid) approach was used for initial base placement and the clash factor and maximum allowed overlap volume were set to 0.6 and 3.5 $Å^3$, respectively.

Selected poses from the initial molecular docking calculations were further optimized using a QM/MM-based potential according to Marion *et al.* (Marion *et al.*, 2017) The QM/MM calculations were performed using the ChemShell suite (Sherwood *et al.*, 2003; Spencer and Walsh, 2006) with the DL_POLY module for the MM-calculations interfacing with Turbomole version 7.1 for the QM-calculations. The MM-region was described applying the parameters from the Amber ff14SB (Maier et al., 2015) force field. The QM-region comprised the side chains of Asn142, His196, Leu197, Asp208, Ser214, Tyr229, Asn262, Lys263, Glu265, and Arg266, and the loop from Asn114 to Gln121, as shown in **Fig. S6**. The QM/MM boundary was located at the nonpolar C_{α}-C_{β} bond of the single side chains and at the N-C_{α} and C_{α}-C' bond of the backbone, respectively. The QM-calculations were performed at the DFT level together with the D3 dispersion correction (Grimme *et al.*, 2010). The TPSS meta-GGA (Tao *et al.*, 2003) functional was applied using RI-J approximation (Eichkorn *et al.*, 1995) on a multigrid m4 (Eichkorn *et al.*, 1997). The SCF convergence criterion was set to 10^{-7} au. The def2-TZVP (Eichkorn *et al.*, 1997; Weigend and Ahlrichs, 2005) basis set was used for the Zn^{2+} ions while the remainder of the QM-

region was described with the def2-SVP (Schäfer *et al.*, 1992; Eichkorn *et al.*, 1997) basis set. An electrostatic embedding scheme was applied to describe the QM/MM boundary, in which link atoms are placed at the boundary and the charges are shifted away and replaced by a dipole on the recipient atom, as implemented in the polarised coupling scheme (*shift)* in ChemShell. The geometry optimisation of all atoms in the QM-region and all direct neighbouring MM-residues was performed using the DL-FIND (Kästner *et al.*, 2009) optimiser.

To summarise, calculations based on the bimetallic enzyme predicted that the carboxylate group of clavulanic acid binds to both metal ions and forms hydrogen bonds with Ser214, Asn254 and Arg257 in the substrate binding pocket of CSR-1_{Trunc} (**Figs 2b**). Once the calculation was performed with single metal occupancy in the α-site (*i.e.* the higher metal affinity site), we observed a stable enzyme-inhibitor complex only in CSR-1_{trunc} and CSR-$1_{trunc,sm}$, indicating the ability of clavulanic acid to displace the β-Zn^{2+} from its low affinity binding site in these two variants of CSR-1. We therefore infer that low metal binding affinity in the β-site is necessary for the observed inhibition by clavulanic acid. Hydrogen bonding to Lys263 may provide additional stabilization of the enzyme-clavulanic acid complex in monometallic CSR-1_{trunc} and CSR-$1_{trunc,sm}$, as this interaction cannot be formed in the double and triple mutants, in which His263 strongly interacts with Zn^{2+} bound in the β site (**Figs 2b**). Since the β-lactam bond in clavulanic acid is prone to hydrolysis (for instance by an attack from an efficient nucleophile such as Lys263) we also investigated interactions between the ring-opened form of the inhibitor with CSR-1 and its variants by docking, using the same approach as employed for the computations with the intact form of clavulanic acid (**Fig. S6**). No significant differences were observed, indicating that clavulanic acid, independent of its state (intact or ring-opened), inhibits the wild-type of CSR-1 and its single mutant.

Table S7| Crystallography parameters for the CSM-1 and its mutants. A third Zn^{2+} ion was observed to bind in the double mutant outside of the α- and β-sites, which we predict is a crystallographic artefact as it would block the active site.

Data collection parameters					
	CSM-1	CSM-1_{trunc}	CSM-$1_{trunc,sm}$	CSM-$1_{trunc,dm}$	CSM-$1_{trunc,tm}$
Resolution Range (Å)	42.29-1.99 (2.04-1.99) [a]	35.16-2.16 (2.22-2.16)	36.90-1.10 (1.12-1.10)	43.75-2.07 (2.52-2.07)	43.77-1.48 (1.50-1.48)
Observations (I>σ(I))	137538 (8753)	57051(17897)	933261 (20130)	126532(8265)	276496 (9478)
Unique reflections (I>σ(I))	18134 (1236)	17930 (1129)	86879 (2205)	19081(1376)	39943 (1589)
Completeness (%)	99.7 (98.3)	95.0 (93.0)	93.9 (48.9)	98.0(87.8)	99.0 (81.0)
Mean <I/σ(I)>	12.0 (3.1)	10.9 (1.3)	12.7 (1.8)	18.4(2.1)	10.1 (2.0)
$^{b}R_{merge}$	0.138 (0.544)	0.095 (0.701)	0.094 (1.254)	0.076(0.689)	0.158 (1.074)
$^{c}R_{p.i.m.}$	0.053 (0.217)	0.082 (0.638)	0.030 (0.417)	0.048(0.441)	0.064 (0.455)
Multiplicity	7.6 (7.1)	3.2 (2.8)	10.7 (9.1)	5.0(6.0)	6.9 (6.0)
Space group	I4	P 3_1 2 1	P 2_1 2_1 2_1	P 2_1 2_1 2_1	P 2_1 2_1 2_1
Unit cell lengths (Å)	a = b = 84.58 c = 74.98	a = b = 74.41 c = 107.47	a = 43.77 b = 68.60 c = 76.25	a = 53.47 b = 74.10 c = 76.10	a = 43.77 b = 69.15 c = 77.52
Unit cell angles (°)	α = β = γ = 90	α = β = 90 γ = 120	α = β = γ = 90	α = β = γ = 90	α = β = γ = 90
**Refinement statistics**					
R_{work}	0.1479	0.1934	0.1700	0.1896	0.1510
R_{free}	0.1808	0.2241	0.1838	0.2202	0.1719
rmsd bond lengths (Å)	0.0025	0.0029	0.011	0.041	0.005
rmsd bond angles (°)	0.58	0.579	1.65	0.549	0.85
[d]Clash score	1.90	0.80	7.60	3.50	0.80
**Ramachandran plot statistics**					
Favoured regions	97.41	96.48	97.96	95.33	98.00
Outlier regions	0.00	0.39	0.00	0.00	0.00
Rotamer outliners	1.45	2.55	3.55	0.51	1.03
PDB code	6DN4	6DQ2	6DQH	6NC5	6DR8

[a]The values in parentheses are for the outer resolution shell. $^{b}R_{merge} = \sum_{hkl}\sum_i |I_i(hkl) - (I(hkl))| / \sum_{hkl}\sum_i I_i(hkl)$. $^{c}R_{p.i.m.} = \sum_{hkl}\left[\frac{1}{[N(hkl)-1]}\right]^{1/2}\sum_i |I_i(hkl) - (I(hkl))| / \sum_{hkl}\sum_i I_i(hkl)$, where $I_i(hkl)$ is the observed intensity and $I(hkl)$ is the average intensity obtained from multiple observations of symmetry-related reflections.
[d] Clashscore is defined as the number of bad overlaps ≥0.4 Å per thousand atoms.

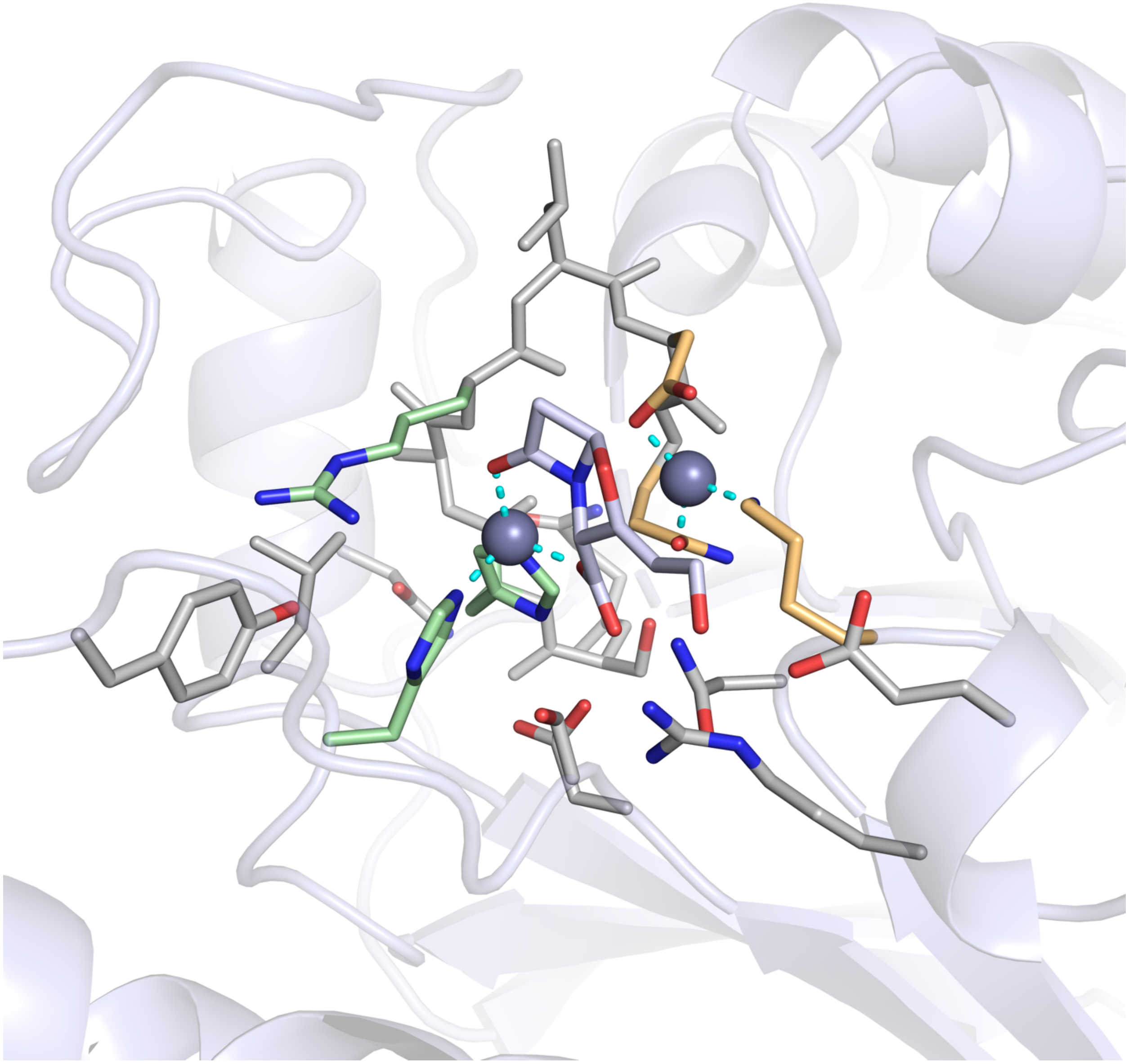

Fig. S7| QM region of CSR-1$_{trunc}$, hydrogen atoms are not shown. Zn^{2+} ions are represented as grey spheres, ions are represented as gray spheres, the carbon atoms of the protein and clavulanic acid are colored in green and grey, respectively, while oxygen and nitrogen atoms are in red and blue.

Supplemental Material References

Adams, P.D., Afonine, P.V., Bunkóczi, G., Chen, V.B., Davis, I.W., Echols, N., Headd, J.J., Hung, L.-W.W., Kapral, G.J., Grosse-Kunstleve, R.W., *et al.* (2010). PHENIX: a comprehensive Python-based system for macromolecular structure solution. Acta Crystallogr. D. Biol. Crystallogr. 66(Pt 2):213-221.

Case, D.A., Betz, R.M., Cerutti, D.S., Cheatham, T.E., III, Darden, T.A., Duke, R.E., Giese, T.J., Gohlke, H., Goetz, A.W., Homeyer, N., *et al.* (2016). AMBER 16 (University of California, San Francisco).

Drawz, S.M., Bethel, C.R., Doppalapudi, V.R., Sheri, A., Pagadala, S.R., Hujer, A.M., Skalweit, M.J., Anderson, V.E., Chen, S.G., Buynak, J.D., *et al.* (2010). Penicillin sulfone inhibitors of class D β-lactamases. Antimicrob. Agents Chemother. 54(4):1414-1424.

Edgar, R.C. (2010). Search and clustering orders of magnitude faster than BLAST. Bioinformatics 26, 2460-2461.

Eichkorn, K., Treutler, O., Öhm, H., Häser, M., and Ahlrichs, R. (1995). Auxiliary basis sets to approximate Coulomb potentials. Chem. Phys. Lett. 240, 283-290.

Eichkorn, K., Weigend, F., Treutler, O., and Ahlrichs, R. (1997). Auxiliary basis sets for main row atoms and transition metals and their use to approximate Coulomb potentials. Theoret. Chem. Acc. 97(1):119-124.

Emsley, P., Lohkamp, B., and Scott, W.G. (2010). F Features and development of Coot. Acta Crystallogr. D. Biol. Crystallog.66 (Pt4), 486-501.

Garcia-Saez, I., Mercuri, P.S., Papamicael, C., Kahn, R., Frere, J.M., Galleni, M., Rossolini, G.M., and Dideberg, O. (2003). Three-dimensional structure of FEZ-1, a monomeric subclass B3 metallo-β-lactamase from *Fluoribacter gormanii*, in native form and in complex with *D*-captopril. *J. Mol. Biol.* 325(4):651-660.

Grimme, S., Antony, J., Ehrlich, S., and Krieg, H. (2010). A consistent and accurate *ab initio* parametrization of density functional dispersion correction (DFT-D) for the 94 elements H-Pu. *J. Chem. Phys.*132(15):154104.

Horsfall, L.E., Izougarhane, Y., Lassaux, P., Selevsek, N., Lienard, B.M., Poirel, L., Kupper, M.B., Hoffmann, K.M., Frere, J.M., Galleni, M., *et al.* (2011). Broad antibiotic resistance profile of the subclass B3 metallo-β-lactamase GOB-1, a di-zinc enzyme. FEBS J. 278(8):1252-1263.

Hou, C.-F.D., Liu, J.-W., Collyer, C., Mitić, N., Pedroso, M.M., Schenk, G. and Ollis, D.L. (2017). Insights into an evolutionary strategy leading to antibiotic resistance. Nat. Sci. Rep. 7: 40357.

Kalyaanamoorthy, S., Minh, B.Q., Wong, T.K.F., von Haeseler, A., and Jermiin, L.S. (2017). ModelFinder: fast model selection for accurate phylogenetic estimates. Nat. Methods 14(6):587-589.

Kästner, J., Carr, J.M., Keal, T.W., Thiel, W., Wander, A., and Sherwood, P. (2009). DL-FIND: An open-source geometry optimizer for atomistic simulations. J. Phys. Chem. *A* 113(43):11856-11865.

Katoh, K., and Standley, D.M. (2013). MAFFT multiple sequence alignment software version 7: improvements in performance and usability. Mol. Biol. Evol. 30(4):772-780.

Leiros, H.-K.S.K., Borra, P.S., Brandsdal, B.O.O., Edvardsen, K.S., Spencer, J., Walsh, T.R., and Samuelsen, O. (2012). Crystal structure of the mobile metallo-β-lactamase AIM-1 from *Pseudomonas aeruginosa*: Insights into antibiotic binding and the role of Gln157. Antimicrob. Agents Chemother. 56(8):4341-4353.

Letunic, I., and Bork, P. (2016). Interactive tree of life (iTOL) v3: an online tool for the display and annotation of phylogenetic and other trees. Nucleic Acids Res. 44(W1):W242-245.

Maier, J.A., Martinez, C., Kasavajhala, K., Wickstrom, L., Hauser, K.E., and Simmerling, C. (2015). ff14SB: Improving the Accuracy of Protein Side Chain and Backbone Parameters from ff99SB. J. Chem. Theor. Comput. 11(8):3696-3713.

Marion, A., Groll, M., Scharf, D.H., Scherlach, K., Glaser, M., Sievers, H., Schuster, M., Hertweck, C., Brakhage, A.A., Antes, I., *et al.* (2017). Gliotoxin biosynthesis: structure, mechanism, and metal promiscuity of carboxypeptidase GliJ. ACS Chem. Biol. 12(7):1874-1882.

McPhillips, T.M., McPhillips, S.E., Chiu, H.J., Cohen, A.E., Deacon, A.M., Ellis, P.J., Garman, E., Gonzalez, A., Sauter, N.K., and Phizackerley, P.R. (2002). Blu-Ice and the Distributed Control System: software for data acquisition and instrument control at macromolecular crystallography beamlines. J. Synchr. Rad. 9(6):401-406.

Moran-Barrio, J., Lisa, M.N., Larrieux, N., Drusin, S.I., Viale, A.M., Moreno, D.M., Buschiazzo, A., and Vila, A.J. (2016). Crystal structure of the metallo-β-lactamase GOB in the periplasmic dizinc form reveals an unusual metal site. Antimicrob. Agents Chemother. 60(10):6013-6022.

Murphy, T.A., Simm, A.M., Toleman, M.A., Jones, R.N., and Walsh, T.R. (2003). Biochemical characterization of the acquired metallo-β-lactamase SPM-1 from *Pseudomonas aeruginosa*. Antimicrob. Agents Chemother. 47(2):582-587.

Nguyen, L.T., Schmidt, H.A., von Haeseler, A., and Minh, B.Q. (2015). IQ-TREE: a fast and effective stochastic algorithm for estimating maximum-likelihood phylogenies. Mol. Biol. Evol. 32(1):268-274.

Otwinowski, Z., and Minor, W. (1997). Processing of X-ray diffraction data collected in oscillation mode. Methods Enzymol. 276:307-326.

Pedroso, M.M., Ely, F., Mitić, N., Carpenter, M.C., Gahan, L.R., Wilcox, D.E., Larrabee, J.L., Ollis, D.L., and Schenk G. (2014). Comparative investigation of the reaction mechanisms of the organophosphate-degrading phosphotriesterases from *Agrobacterium radiobacter* (OpdA) and *Pseudomonas diminuta* (OPH). J. Biol. Inorg. Chem. 19:1263-1275.

Pedroso, M.M., Ely, F., Carpenter, M.C., Mitić, N., Gahan, L.R., Ollis, D.L., Wilcox, D.E., and Schenk G. (2017). Calorimetric characterization of metal ion binding to the binuclear active site of Glycerophosphodiesterase (GpdQ) from *Enterobacter aerogenes*. Biochemistry 56:3328-3336.

TURBOMOLE V7.1 (2016) A development of University of Karlruhe and Forschungzentrum Kahlruhe GmbH. 1989-2007. TURBOMOLE available http:www.turbomole.com.

Rarey, M., Kramer, B., Lengauer, T., and Klebe, G. (1996). A Fast flexible docking method using an incremental construction algorithm. *J. Mol. Biol.* 261(3):470-489.

Schäfer, A., Horn, H., and Ahlrichs, R. (1992). Fully optimized contracted Gaussian basis sets for atoms Li to Kr. J. Chem. Phys. 97(4):2571-2577.

Segel, I.H. (1993). Enzyme kinetics: Behavior and analysis of rapid equilibrium and steady-state enzyme systems (United States of America, John Wiley & Sons).

Selleck, C., Monteiro Pedroso, M., Wilson, L., Krco, S., Gianna Knaven,E., Miraula, M., Mitić, N., Larrabee, J., Brück,T., Clark, A., Guddat, L., Schenk, G. (2020). Structure and mechanism of potentbifunctional β-lactam- and homoserine lactone-degrading enzymes from marine microorganisms. BioRxiv, https://doi.org/10.1101/2020.03.24.006742.

Selleck, C.L., J. L., Harmer, J.; Guddat, L. W.; Mitić, N.; Helweh, W.; Ollis, D. L.; Craig, W. A.; Tierney, D. L.; Pedroso, M. M.; Schenk, G. (2016). AIM-1: An antibiotic-degrading metallohydrolase that displays mechanistic flexibility. Chem. Eur. J. *22*(49):17704-17714.

Sherwood, P., de Vries, A.H., Guest, M.F., Schreckenbach, G., Catlow, C.R.A., French, S.A., Sokol, A.A., Bromley, S.T., Thiel, W., Turner, A.J*., et al.* (2003). QUASI: A general purpose implementation of the QM/MM approach and its application to problems in catalysis. J. Mol. Struct. Theochem. 632(1):1-28.

Spencer, J., Clarke, A.R., and Walsh, T.R. (2001). Novel mechanism of hydrolysis of therapeutic beta-lactams by Stenotrophomonas maltophilia L1 metallo-beta-lactamase. J. Biol. Chem. 276(36):33638-33644.

Tao, J., Perdew, J.P., Staroverov, V.N., and Scuseria, G.E. (2003). Climbing the density functional ladder: nonempirical meta-generalized gradient approximation designed for molecules and solids. Phys. Rev. Lett. 91(14):146401.

Ullah, J.H., Walsh, T.R., Taylor, I.A., Emery, D.C., Verma, C.S., Gamblin, S.J., and Spencer, J. (1998). The crystal structure of the L1 metallo-β-lactamase from *Stenotrophomonas maltophilia* at 1.7 A resolution. J. Mol. Biol. 284(1):125-136.

Vella, P., Miraula, M., Phelan, E., Leung, E.W., Ely, F., Ollis, D.L., McGeary, R.P., Schenk, G., and Mitic, N. (2013). Identification and characterization of an unusual metallo-β-lactamase from *Serratia proteamaculans*. J. Biol. Inorg. Chem. 18(7):855-863.

Weigend, F., and Ahlrichs, R. (2005). Balanced basis sets of split valence, triple zeta valence and quadruple zeta valence quality for H to Rn: Design and assessment of accuracy. *Phys. Chem. Chem. Phys.* 7(18):3297-3305.
